# Pedicled Thoracodorsal Artery Perforator Flap in Breast Reconstruction: Clinical Experience

**Published:** 2009-07-21

**Authors:** Neta Adler, Iris A. Seitz, David H. Song

**Affiliations:** ^a^ Division of Pediatric Plastic Surgery, The Children's Memorial Hospital, Chicago, Ill; ^b^ Section of Plastic and Reconstructive Surgery, University of Chicago, Chicago, Ill

## Abstract

**Background:** The thoracodorsal artery perforator (TDAP) flap has been described for reconstruction of the head and neck, trunk and extremities. Yet, its use as a pedicled flap in breast reconstruction has not gained wide popularity and has not been widely documented, especially not for complete breast reconstruction or in combination with expanders or permanent implants. The authors present their clinical experience with the thoracodorsal artery perforator flap in breast reconstruction. **Methods:** From February 2007 to February 2009, eighteen patients had breast reconstruction utilizing a TDAP flap. Retrospective analyzes of patient characteristics, breast history, clinical indications, complications and outcomes were performed. The follow-up period ranged from 1 to 17 months. **Results:** Eleven patients had complete breast reconstruction using a TDAP flap with simultaneous insertion of an expander or implant. Four cases were partial reconstruction to gain additional volume after previous breast reconstruction and the 3 other cases were reconstruction after lumpectomy. All flaps survived. Two case required evacuation of hematoma. One case had late extrusion of the expander after expansion in the previously irradiated tissue, requiring expander removal. There were no donor site complications. **Conclusions:** The TDAP flap has proven to be a reliable flap with minimal donor site morbidity. Patients who had radiation treatment prior to reconstruction with pronounced radiated chest skin changes might still benefit from additional tissue from the LD muscle.

In 1995, Angrigiani et al^1^ first described the use of a cutaneous island of the latissimus dorsi (LD) flap without the muscle based on a single perforator of the thoracodorsal artery for lower extremity reconstruction. Since then the thoracodorsal artery perforator (TDAP) flap has been described for reconstruction of the head and neck, trunk, and extremities.

Yet, its use as a pedicled flap in breast reconstruction has not gained wide popularity and has not been widely documented, especially not for complete breast reconstruction or in combination with expanders or permanent implants.^2^–^4^ A recent study by Hamdi et al^5^ presented 4 cases of combined TDAP with prosthesis for breast reconstruction with good results in primary reconstruction and after failure of other techniques.

The role of TDAP flap in breast reconstruction still needs to be established. Its potential advantages or disadvantages, when compared with latissimus dorsi flap reconstruction in combination with implant or expander, are yet in question. The theoretical advantage of sparing the LD muscle and thus perhaps reducing the donor site morbidity is appealing; however, this concept still needs to be studied in prospective controlled studies. Moreover, in radiated patients with complete breast reconstruction, coverage of the lower part of the prosthesis with cutaneous flap might not be sufficient and the use of muscle flap to lower the rates of prosthesis exposure could be necessary. Capsular contracture rates might be higher if muscle coverage is not provided. Also, the TDAP flap dissection might be more demanding for the reconstructives surgeon, requiring experience in microsurgical techniques.

We describe here our clinical experience with the TDAP flap in reconstruction of breast.

## ANATOMY

The main pedicle of the TDAP flap is the thoracodorsal artery, which originates from the subscapular axis. Two anatomical landmarks based on anatomical studies of fresh cadavers were presented previously for guidance for flap elevation. The first landmark described by Angrigiani et al^1^ is the point 8 cm below the posterior axillary fold and 2 cm behind the lateral border of the LD muscle where the proximal skin perforator derived from the descending branch of the thoracodorsal artery exits the muscle into the subcutaneous tissue. The second landmark described by Heitmann et al^6^ is the site of the thoracodorsal artery bifurcation point into lateral or descending branch and horizontal or medial branch and is located on the deep surface of the LD muscle 3 to 6 cm inferior to the inferior scapular tip and 1 to 4 cm medial to the lateral free margin of the muscle. In this study, all the cutaneous perforators were found within a distance of 8 cm distal to the neurovascular branching point. The lateral branch usually gives 1 to 4 cutaneous perforators. More recent anatomical studies showed some variation in the location of the branching point and the perforators.^7^,^8^ These anatomical variations might be explained partially by the difference between using cadaveric and live subjects for different studies and by marking the site of the skin perforators in different positions–sitting position, lateral decubitus, or supine. The arm position might also change the relationship between the skin perforators and other anatomical landmarks due to relaxation or stretching of the overlying skin and movement of the scapular tip. Preoperative color Doppler was suggested to visualize the perforators and the adjacent anatomical structures because of the uncertainty of the anatomic landmarks.^7^ The use of Doppler flowmetry rather than color Doppler imaging can be misleading because it does not easily distinguish perforating vessels from main axial vessels. A direct cutaneous branch arising from the thoracodorsal artery before its branching point or from the subscapular or axillary artery can be found in 55% to 81% of the cases. This branch does not pierce the latissimus muscle but rounds the lateral edge of the muscle to contribute to the blood supply of the lateral thoracic skin and subcutaneous tissue.^6^

## PATIENTS AND METHODS

In the time period from February 2007 to February 2009, 18 patients had breast reconstruction with TDAP–11 cases of complete reconstruction and 7 cases of partial reconstruction. The patient characteristics and clinical indications are summarized in Table 1.

### Surgical technique

**Figure 1:**
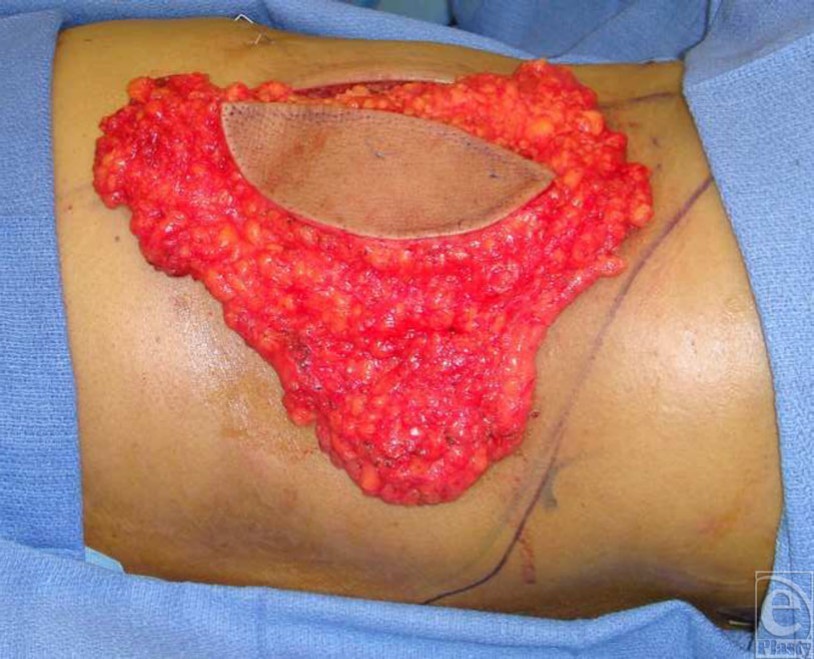
Thoracodorsal artery perforator flap elevated. Maximum fat should be included in the dissection.

**Figure 2:**
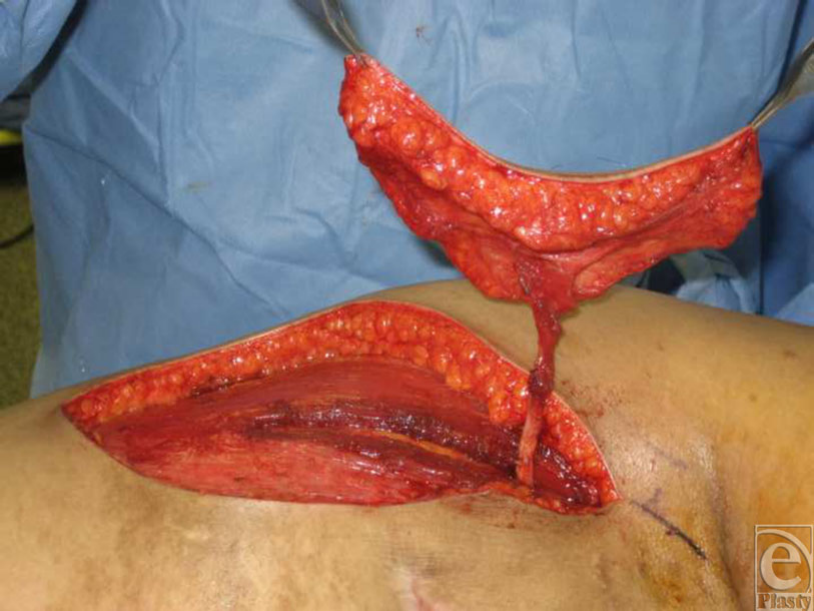
Thoracodorsal vessels dissected until enough length is achieved to allow insetting of the flap with no tension.

The patient is placed in the lateral position. The arm is abducted 90° as for harvesting a classical LD flap. The flap dimensions are marked in this position on the basis of the defect size and the ability to close the donor site primarily. The latter can be estimated using the skin pinch test. The flap's central point is located 8 cm below the posterior axillary fold and 2 cm behind the anterior margin of the LD muscle. The flap can be designed in several deferent ways, depending on the patient's preference for the direction of the donor site scar–its long axis can be directed vertically so that the donor site scar would be placed eventually in the continuation of the posterior axillary line and would not protrude to the back or chest or more horizontally underneath the bra line. The dissection begins from the anterior side in a suprafascial plane. The dissection must be beveled to include a maximum of fat (Fig 1). Once a *palpable* pulsating perforator is found, we proceed to dissect the perforator intramuscular to the descending branch. If no palpable pulsating perforator is found, we dissect a small cuff of muscle to include a few smaller perforators. The vascular pedicle is dissected until enough length is achieved to allow placement of the flap at the defect site with no tension (Fig 2). The nerve that is running along with the pedicle is dissected free and spared. A tunnel is made between the donor site and the defect, and then the flap is interpolated into the defect and secured. The donor site is closed. For flap insetting the patient is placed in a supine position to compare symmetry of both breasts. Both breasts are prepped and draped again and the flap is then inset. If an implant or expander is needed for additional volume, a pocket is created in the subpectoral plane to accommodate their placement and the flap is inset above the pectoralis muscle. After insetting, appearance and symmetry are checked with the patient in a sitting position.

## RESULTS

**Figure 3:**
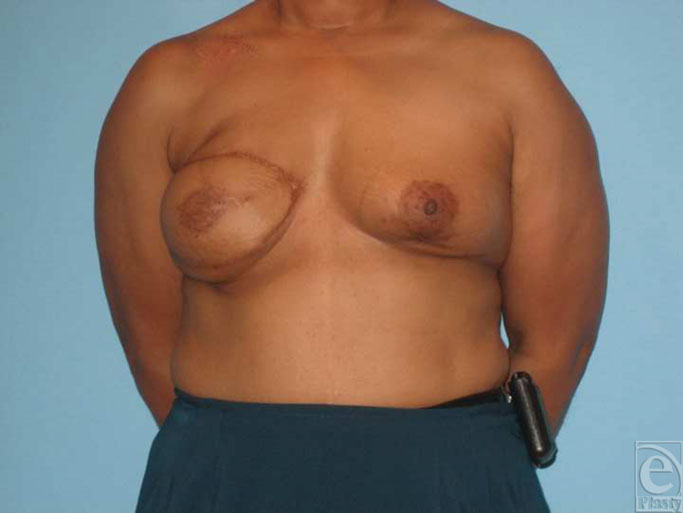

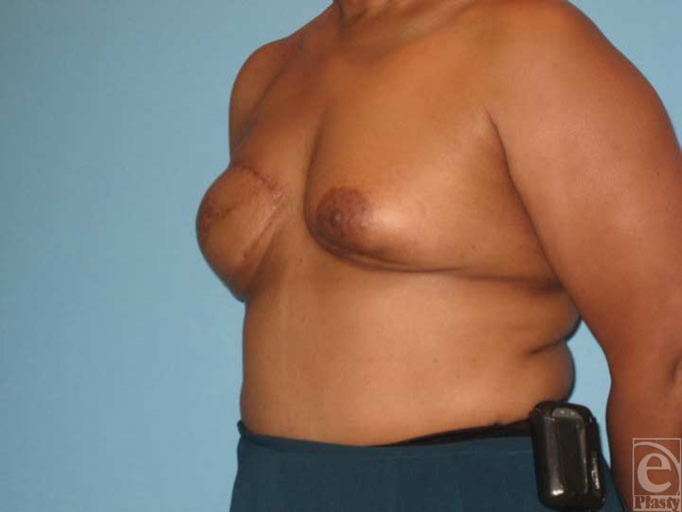

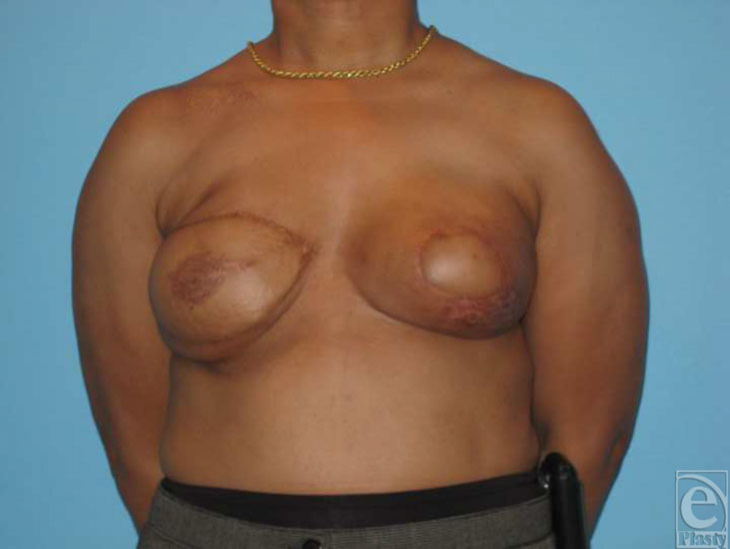

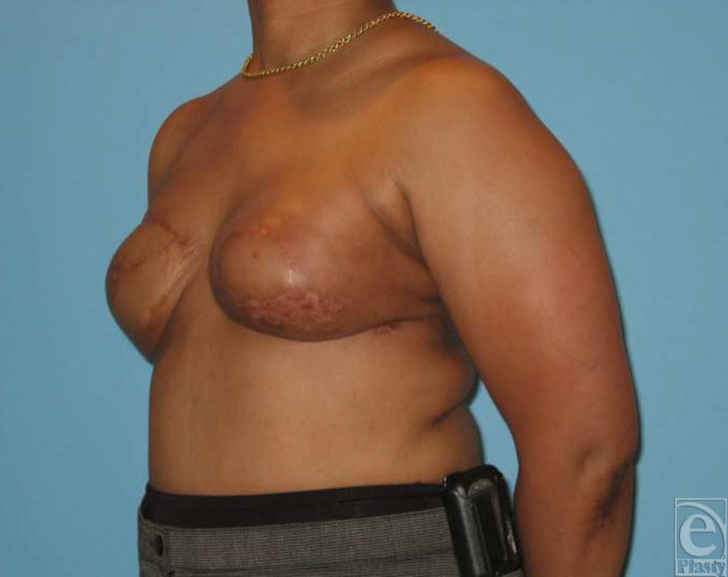

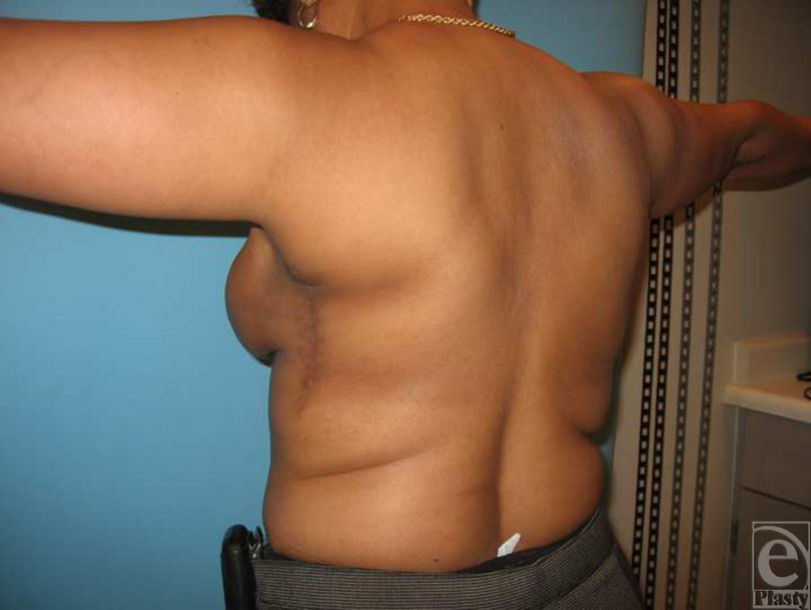
Patient 5. (a and b) Preoperative view of a 50-year-old woman status post–right breast reconstruction with TRAM flap in another hospital and biopsy of right breast with positive margins for carcinoma. (c and d). Three months after immediate left mastectomy and reconstruction with TDAP flap and simultaneous implant insertion. (e). Donor site 3 months postoperative.

**Figure 4:**
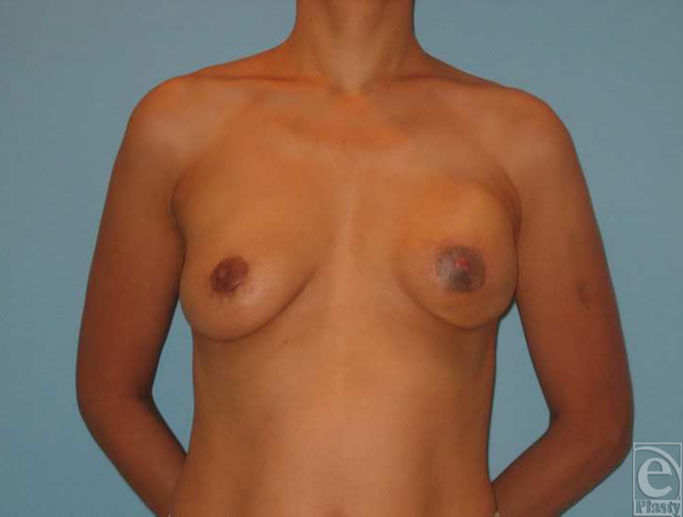

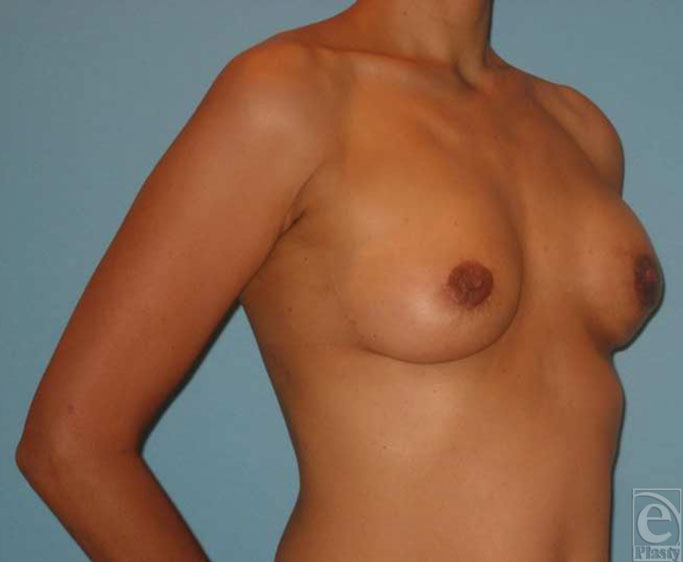

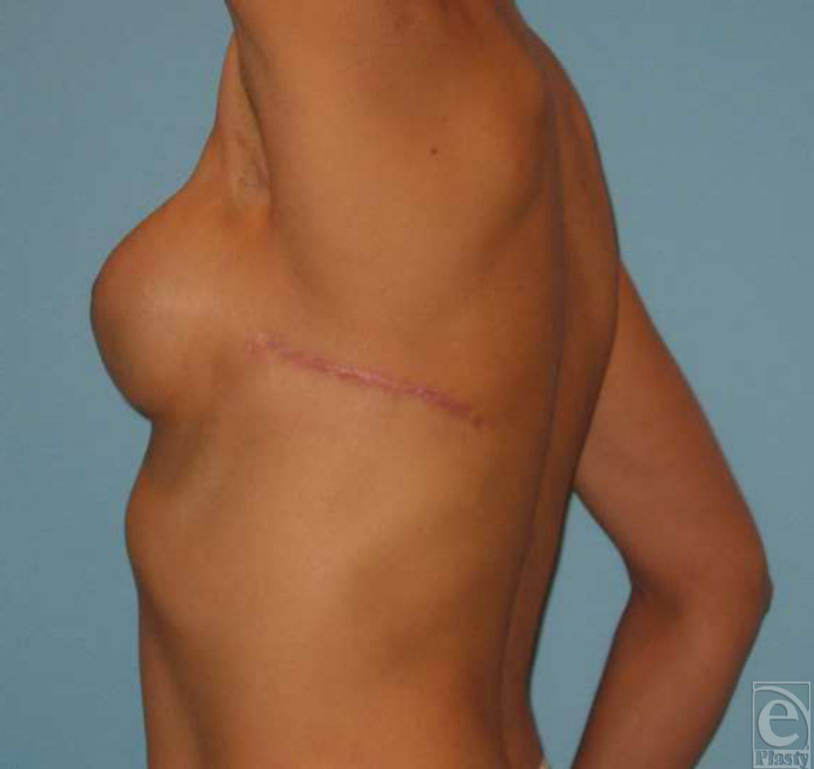
Patient 7. (a and b) A 40-year-old woman, 5 months after left skin sparing mastectomy and immediate reconstruction with TDAP flap and implant insertion. (c) Donor site 5 months postoperative.

Eighteen patients were operated on using the technique described. Procedure type, complications, and follow-up are summarized in Table 1. In 11 cases of complete breast reconstruction, an expander or implant was inserted as well. Two of them were salvage procedures for breakdown after insertion of an implant, 6 patients had immediate reconstruction, 4 had delayed reconstruction, and 1 case was bilateral reconstruction delayed on one side and immediate on the other side. Four cases were partial reconstructions to gain additional volume after previous breast reconstruction and the other 3 cases had reconstruction after lumpectomy. All flaps survived (Figs 3 and 4). Two cases required evacuation of hematoma in the operating room. One case had late extrusion of the expander probably due to combination of overexpansion in irradiated tissue. The expander was removed and the patient had contralateral mastectomy and reconstruction with bilateral deep inferior epigastric perforator flap. There was one case of expander removal due to infection that was not resolved with antibiotic treatment. There were no donor site complications. The follow-up period ranged from 1 to 17 months.

## DISCUSSION

The pedicled TDAP flap can be used for partial breast reconstruction after lumpectomy or as additional bulk for previously reconstructed breast or for complete breast reconstruction with expander or implant. However, the indications to choose the TDAP flap over the classical LD flap remain unclear, since the harvesting of the TDAP flap is more technically demanding and there are no studies that compare donor site morbidity between these flaps. Still, we believe there are a few important advantages to this flap. First, several studies showed that after transfer of the LD muscle, shoulder strength and/or the range of motion deteriorate.^9^,^15^ These studies have several limitations; most of them are retrospective and only 2 evaluate pedicled LD flap. Only a few studies used isokinetic measurements. Other contradicting studies do not show any long-term morbidity after LD muscle harvesting for breast reconstruction.^16^ Also, a recent prospective study showed maintained LD strength between operated side and nonoperated side after harvesting of TDAP flap and no muscle atrophy with average follow-up of 19 months.^17^ Although lack of prospective studies comparing donor site morbidity between LD flap and TDAP flap, in a certain group of young athletic patients it is probably worthwhile to preserve the muscle. In addition, preserving the LD muscle is probably less painful postoperatively. Second, harvesting of the LD muscle results in high seroma rate of the donor site.^18^–^20^ None of our cases had donor site seromas. Third, the aesthetic result might be somewhat superior with the TDAP flap because of better preservation of the posterior axillary fold. From our experience, harvesting of the TDAP flap is predictable if one uses microsurgical techniques and takes a cuff of muscle in the event that there are no large pulsating perforators. Hamdi et al^4^ presented an algorithm for choosing pedicled flaps in which the amount of LD muscle harvested with the flap depends on the number and quality of the perforators found. If a good size pulsatile perforator is found, a TDAP flap is chosen. If there are no large perforators but pulsatile, a small piece of LD muscle is incorporated within the flap. A larger segment of LD muscle is designed if there are multiple tiny perforators that are nonpulsatile. Our cutoff for converting from true perforator flap to muscle sparing was based on the presence of palpable pulsatile perforator. Based on this criterion, 20% of our cases had to be converted to muscle sparing LD flap. In a recently published study also by Hamdi et al,^21^ the TDAP flap was used in 99 patients for a variety of indications, mostly partial breast reconstruction with low complication rate.

The use of the TDAP-flap combined with an expander or implant is a new tool for complete breast reconstruction. Four cases published by Hamdi et al^5^ of combined TDAP flap with implant did not show any compromise of the blood supply to the flap as one may expect because of pressure on the perforator. However, they suggested adding a small amount of muscle when the dissected perforator entered the flap near the center of the skin paddle and thus will be near the implant after insetting. In the 8 cases of complete breast reconstruction described here, the healing of the flap was good although 1 case resulted in late expander removal due to exposure probably as a result of overexpansion in irradiated tissue. Since the lower part of the implant or expander is covered only by the TDAP flap, it might be possible that in radiated patients additional tissue from LD muscle would help preventing extrusion. The decision of placing an expander or permanent implant is based on clinical judgment considering the size of the pocket relative to the breast size desired and risk factors such as smoking and radiation.

Capsular contracture around the implant or expander was Becker grade I and II. Additional series with longer follow-up is needed to compare the capsular contracture rates between TDAP and LD flaps.

Another application of the TDAP flap that we have found to be useful in breast reconstruction is for further refinements of the aesthetic results when additional volume is required for reconstruction of the nipple-areola complex area. The flap can be placed in the area of the planned nipple-areola complex and thus create the slight natural bulge that otherwise is hard to achieve.

In conclusion, the TDAP flap has mostly replaced the LD flap in our practice for autologus partial breast reconstruction or for complete breast reconstruction in women with contraindication or who are unwilling to have reconstruction from abdominal tissue. It has proven to be a reliable flap with minimal donor site morbidity. Patients who had radiation treatment prior to reconstruction with pronounced radiated chest skin changes might still benefit from additional tissue from the LD muscle.

## Figures and Tables

**Table 1:** Patients' characteristics, clinical indications for flap reconstruction, procedure done, complications, and follow-up time*

Case	Age	Indication	Risk factors	Procedure	Complications	Follow-up time, mo
1	57	Additional bulk to previously reconstructed breast with TRAM flap	None	Delay of TDAP flap–TDAP flap (2 wk after delay)	Hematoma, Small skin dehiscence, completely healed conservatively	17
2	52	Additional bulk to previously reconstructed breast with implant	Smoking	TDAP flap	None	1
3	65	Additional bulk to previously reconstructed breast with intercostal artery perforator flap	Preoperative radiation	TDAP flap	None	12
4	43	Delayed complete breast reconstruction	Preoperative radiation	TDAP flap and expander insertion	Extruded expander	16
5	50	Immediate complete breast reconstruction	None	TDAP flap and implant insertion	Partial necrosis of mastectomy breast flap laterally treated conservatively	12
6	43	Exposure of implant	Preoperative radiation	TDAP flap and expander insertion	None	2.5
7	47	Immediate complete breast reconstruction	None	TDAP flap and implant insertion	None	5
8	50	Breakdown and expose of AlloDerm after replacement to permanent implant	Preoperative radiation	TDAP flap (implant was inserted in a pervious operation)	None	9
9	69	Delayed breast reconstruction	Preoperative radiation	TDAP flap and implant insertion	None	3
10	40	Immediate complete breast reconstruction	None	TDAP flap and implant insertion	None	4
11	40	Immediate complete breast reconstruction	None	TDAP flap and implant insertion	None	9
12	58	Early lumpectomy	Postoperative radiation	TDAP flap	None	5
13	60	Early lumpectomy	None	TDAP flap	None	1
14	58	Delayed complete breast reconstruction	None	TDAP flap and expander insertion	Expander removal due to infection	2
15	55	Delayed complete breast reconstruction	None	TDAP flap and expander insertion	None	1
16	52	Immediate lumpectomy	None	TDAP flap	None	1
17	53	Additional bulk to previously reconstructed breast with implant	None	TDAP flap (implant was inserted in a pervious operation)	Hematoma	1
18	53	Bilateral complete reconstruction, immediate on one side and delayed on the other side	Preoperative radiation to the delayed breast reconstruction	Bilateral TDAP flap and expander insertion	None	1

*TDAP indicates thoracodorsal artery perforator; TRAM, transverse rectus abdominis myocutaneous flap.
